# Residential Traffic and Children’s Respiratory Health

**DOI:** 10.1289/ehp.10735

**Published:** 2008-05-27

**Authors:** Janice J. Kim, Karen Huen, Sara Adams, Svetlana Smorodinsky, Abby Hoats, Brian Malig, Michael Lipsett, Bart Ostro

**Affiliations:** 1 Office of Environmental Health Hazard Assessment, Air Pollution Epidemiology Section, Oakland, California, USA; 2 Environmental Health Sciences, School of Public Health, University of California, Berkeley, California, USA; 3 Environmental Health Investigations Branch, California Department of Public Health, Richmond, California, USA; 4 Impact Assessment, Inc., La Jolla, California, USA

**Keywords:** air pollution, asthma, bronchitis, children, respiratory health, traffic

## Abstract

**Background:**

Living near traffic has been associated with asthma and other respiratory symptoms. Most studies, however, have been conducted in areas with high background levels of ambient air pollution, making it challenging to isolate an independent effect of traffic. Additionally, most investigations have used surrogates of exposure, and few have measured traffic pollutants directly as part of the study.

**Objective:**

We conducted a cross-sectional study of current asthma and other respiratory symptoms in children (*n* = 1,080) living at varying distances from high-traffic roads in the San Francisco Bay Area, California, a highly urbanized region characterized by good regional air quality due to coastal breezes.

**Methods:**

We obtained health information and home environmental factors by parental questionnaire. We assessed exposure with several measures of residential proximity to traffic calculated using geographic information systems, including traffic within a given radius and distance to major roads. We also measured traffic-related pollutants (nitrogen oxides and nitrogen dioxide) for a subset of households to determine how well traffic metrics correlated with measured traffic pollutants.

**Results:**

Using multivariate logistic regression analyses, we found associations between current asthma and residential proximity to traffic. For several traffic metrics, children whose residences were in the highest quintile of exposure had approximately twice the adjusted odds of current asthma (i.e., asthma episode in the preceeding 12 months) compared with children whose residences were within the lowest quintile. The highest risks were among those living within 75 m of a freeway/highway. Most traffic metrics correlated moderately well with actual pollutant measurements.

**Conclusion:**

Our findings provide evidence that even in an area with good regional air quality, proximity to traffic is associated with adverse respiratory health effects in children.

Epidemiologic studies have linked proximity to busy roads with adverse health outcomes, including respiratory symptoms, asthma, adverse birth outcomes, and cardiopulmonary mortality (Brunekreef et al. 1997; Hoek et al. 2002; Wilhelm and Ritz 2003). Methods for estimating exposures to traffic pollutants have included neighborhood- or school-based estimates of traffic (Brunekreef et al. 1997; Kim et al. 2004), distance to freeways or busy roads (Gauderman et al. 2005), presence of a busy road within a given buffer (Venn et al. 2001), and traffic density within a given radius (English et al. 1999; Wilhelm and Ritz 2003). More recent studies have used geographic information systems (GIS) to estimate traffic exposure metrics. However, few have evaluated these GIS-based traffic metrics against measured traffic-related pollutants (Brauer et al. 2007; Gauderman et al. 2005; Hoek et al. 2002; Nicolai et al. 2003). Additionally, many of these studies were conducted in areas with moderate or high levels of regional air pollution.

We conducted the East Bay Children’s Respiratory Health Study (EBCRHS) in the San Francisco Bay Area, California, a highly urbanized region of the United States where traffic is the major source of air pollution. This region ranks among the top four metropolitan areas with the worst traffic congestion in the United States (Schrank and Lomax 2005). However, the area experiences relatively good regional air quality due to onshore breezes. Thus, in contrast to most major metropolitan areas in the United States, there are only occasional exceedances of the federal ozone or fine particulate matter [particles ≤2.5 μm in diameter (PM_2.5_)] 24-hr standard. This allowed us to examine the impacts of local variations in traffic in the absence of significant levels of background ambient pollution.

In the first phase of this study, we found modest but statistically significant associations between measured traffic pollutants and recent episodes of asthma and bronchitis. In that analysis, we measured traffic-related pollutants at schools as indicators of neighborhood air pollution levels, which we used to estimate children’s exposure to traffic emissions (Kim et al. 2004).

In this analysis, we sought to refine exposure estimates using GIS-derived traffic measures at the children’s residences and to evaluate associations between residential proximity to traffic and respiratory health outcomes for the study population. We then evaluated whether traffic pollutants measured at the schools were independently associated with the health outcomes. We also evaluated the correlation of GIS-derived traffic proximity metrics and vehicular emissions for a subset of households using measurements of traffic-related pollutants (total nitrogen oxides and nitrogen dioxide).

## Materials and Methods

### Study design and health assessment

The EBCRHS study design has been described elsewhere (Kim et al. 2004; Singer et al. 2004). Briefly, in 2001 we recruited students in grades 3–5 from 10 neighborhood schools located at various distances from major roadways. No residences were near major stationary sources of pollution.

We obtained respiratory health outcomes by parental questionnaire. Main outcomes examined were *a*) current asthma [physician-diagnosed asthma at some time in the past (ever asthma) plus “an episode of asthma” or “wheezing” in the preceding 12 months] and *b*) bronchitis symptoms in the preceding 12 months (being told by a doctor the child had bronchitis or persistent cough or phlegm production in the preceding 12 months). Additional questionnaire data included demographics, familial history of asthma, home and environmental factors, and the child’s activity patterns. Parents gave written informed consent before the study. The Committee for the Protection of Human Subjects of the California Health and Human Services Agency reviewed and approved the study protocol. We have complied with all applicable requirements of the California Health and Human Services Agency.

Other sources of data for this study included *a*) California Department of Transportation (Sacramento, CA) annual average daily traffic (AADT) for 2001 and road classification data for all freeways, highways, and major (nonlocal) roads; *b*) meteorologic data for Oakland and Hayward airports (Western Region Climate Center, Reno, NV); and *c*) traffic pollutant measurements conducted for this project. For additional details on study design and methods, see Supplemental Material (http://www.ehponline.org/members/2008/10735/suppl.pdf).

### Exposures to traffic pollution

We geocoded residential addresses of study participants and determined residential proximity to traffic using metrics that previous studies found associated with adverse health outcomes (English et al. 1999; Gauderman et al. 2005; Gunier et al. 2003). We conducted GIS analyses using ArcGIS 8.3 (Environmental Systems Research Institute, Redlands, CA). We calculated traffic metrics for our study participants, as described in Table 1 [see Supplemental Material (http://www.ehponline.org/members/2008/10735/suppl.pdf)]. These measures used data on traffic counts on nearby roads, distances from home to road, and/or road length within a given radius of the home.

To explore the influence of wind direction, we also calculated a three-level ordinal variable incorporating both residential proximity to a freeway/highway and location upwind or downwind of a freeway: *a*) ≤300 m of a freeway/highway and downwind; *b*) ≤300 m of a freeway/highway and upwind; and *c*) > 300 m from a freeway/highway, regardless of wind direction (reference group). Freeways and highways in the study area generally run north/south, and prevailing winds are from the west. Therefore, we designated locations east of the freeways as downwind, and those west of the freeways as upwind. A few residences (*n* < 10) located upwind of a major freeway and downwind of an intersecting smaller highway were designated as downwind.

### Measured traffic pollutants versus GIS-based traffic metrics

NO_x_ and NO_2_ are good indicators of nearby traffic (Rodes and Holland 1981; Singer et al. 2004). In our earlier study (Kim et al. 2004), we had measured NO_x_, NO_2_, particulate matter ≤10 μm in aerodynamic diameter (PM_10_), PM_2.5_, and black carbon (BC) at 10 school sites. In this expanded monitoring study, we measured only NO_x_ and NO_2_ because of logistical and financial constraints. We measured outdoor concentrations of NO_x_ and NO_2_ using Ogawa passive diffusion samplers (Ogawa & Co., Inc., Pompano Beach, FL) deployed for a 1-week period at 52 locations in the study area (10 schools, 41 student residences or neighborhood locations, and 1 regional air monitor), as previously described (Singer et al. 2004). These sites were at varying distances upwind or downwind of a major freeway. We determined locations of the samplers using a global positioning system device. For each location, we determined GIS-based traffic metrics and upwind/downwind status as described above. Initial NO_x_ emissions in traffic exhaust are primarily in the form of nitric oxide, which subsequently reacts with ambient oxidants to form NO_2_. Thus, we estimated the concentration of NO by the difference NO = NO_x_ − NO_2_.

We evaluated the relationships between NO_x_, NO_2_, and NO and GIS-based traffic metrics at the same locations using Spearman’s correlation coefficient. We used univariate analysis to assess the relationship between NO_x_ and distance to a freeway or the natural logarithm of distance to a freeway. To evaluate the influence of wind direction, we added an interaction term between downwind and natural log of distance. We tested whether median pollutant levels differed by the categories > 300 m, ≤300 m downwind, and ≤300 m upwind using the Wilcoxon rank-sum test (α adjusted for Bonferroni inequality).

### Associations of traffic exposure with health outcomes

We examined associations between each traffic measure and health outcomes using multivariate logistic regression. We identified potential confounders and effect modifiers via parental responses to questionnaires distributed through the children’s schools. For model development, we evaluated risk factors that previous studies showed to be predictors of respiratory disease, including demographic variables (e.g., race/ethnicity, parental education, household income), host factors (e.g., family history of asthma), and home environmental factors (e.g., home exposure to environmental tobacco smoke, household mold), as previously described (Kim et al. 2004). We identified initial variables using univariate regressions, with variable retention if *p* ≤0.15. We used stepwise logistic regression to identify individual-level covariates that were best associated with health outcomes in multivariate models. Using stepwise backward elimination, we retained covariates that changed regression estimates of traffic metrics by > 10% in the final model. We calculated adjusted odds ratios (ORs) and 95% confidence intervals (CIs) for each quintile of traffic and for the 90th percentiles based on the metric’s distribution for the study population. We explored possible dose–response relationships across quintiles by testing for trend using quintiles as categorical variables (Jewell 2004).

We also calculated odds for a simpler traffic metric, distance of residence to major road, using either linear- or log-distance. For distance to major road, we evaluated risks of current asthma or bronchitis for the categories ≤75 m, > 75 and ≤150 m, > 150 and ≤300 m, and > 300 m, based on results of previous studies demonstrating that elevated pollutant concentrations near freeways decreased to background levels by around 150–300 m downwind (Rodes and Holland 1981; Zhu et al. 2002a, 2002b). We looked for associations between respiratory symptoms and residential proximity to other principal arterial roads, as classified by federal standards [see Supplemental Material (http://www.ehpon-line.org/members/2008/10735/suppl.pdf)], after restricting our analysis to those who did not live within 150 m of a freeway/highway. We also evaluated traffic metrics incorporating wind direction. We conducted several sensitivity analyses related to exposure assessment, including *a*) evaluating distance-Gaussian-weighted traffic density (another measure of traffic density), as proposed by English et al. (1999) and Wilhelm and Ritz (2003); *b*) increasing the buffer radius of traffic measures to 300 m; *c*) restricting the sample to those who had lived at their current residence for at least 1 year; and *d*) determining whether traffic pollution from both home and school were independently associated with respiratory morbidity, which we tested by including both exposure locations in the regression model, with school exposures measured using either the traffic-based metrics or the pollution measurements taken at the schools, as in Kim et al. (2004). Additionally, we evaluated associations using a different definition of current asthma (told by a doctor that the child had asthma in the preceding 12 months). Finally, we conducted stratified analyses to explore whether associations between residential proximity to traffic and health outcomes differed by sex and family history of asthma.

We conducted all statistical analyses using SAS, versions 8.2 and 9.1 (SAS Institute Inc., Cary, NC) or STATA version 8 (Stata Corp., College Station, TX).

## Results

### Study population and demographics

More than 70% of students who received questionnaires participated in the study (1,111 of 1,571). We were able to geocode 1,086 (98%) participant addresses. Among these participants, we excluded four because they resided in a neighboring county for which traffic data were not readily available, and two because they had cystic fibrosis. The final study population consisted of 1,080 participants.

Table 2 summarizes data on demographics, home environmental factors, health status, and traffic exposures. The study population was of lower economic status and more racially and ethnically diverse than the general population of California, reflecting the demographics of the study area. More than 30% of household incomes were at or below the federal poverty level. Sixteen percent of study participants lived within 100 m of a major road (principal artery, expressway, highway, or freeway); 5% lived within 100 m of a freeway/highway. This indicates that a considerable proportion of children in our study resided in close proximity to busy roads [for additional data on distribution of traffic exposures, see Supplemental Material (http://www.ehponline.org/members/2008/10735/suppl.pdf)]. Our population was considerably mobile; only 30% had lived at the same address since before 2 years of age; 19% had lived at their current address for less than 1 year.

### Measured traffic pollutant versus GIS-based traffic metric

Pollutant measurements took place in spring 2001 during one of two nonconsecutive weeks. We did not monitor all sites simultaneously because of resource constraints, but we monitored 11 sites during both weeks. These 11 sites showed no statistical difference between the pollutant concentrations. This allowed us to combine data from both weeks into a single data set.

**Figure 1:**
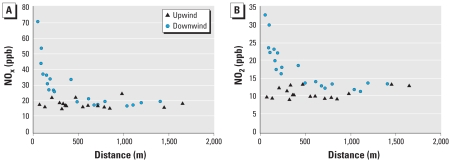
Concentrations of NO_x_ (*A*) and NO_2_ (*B*) as a function of distance to freeway/highway. Data are for week 1.

Table 3 shows correlations between measured NO_x_, NO_2_, and NO and traffic metrics based on 52 samples. Most traffic metrics were better correlated with NO_x_ and NO compared with NO_2_. Traffic density and maximum AADT were significantly correlated with pollutants and explained between 35% and 60% of the variability in NO_x_ and NO. Correlations between NO_2_ levels and traffic metrics (other than distance to freeway/highway) were significant only for metrics using 300-m buffers. Plots of NO_x_ and NO_2_ versus distance to the closest freeway/highway suggest that *a*) levels differ for a given distance depending on whether the location was upwind or downwind of the freeway, and *b*) the pollutant concentration decayed exponentially downwind (Figure 1). Consistent with the observed exponential decay, the log of distance from the freeway/ highway to a residence was a better predictor of NO_x_ than the linear distance in univariate regressions. An interaction term between log-distance and an indicator of wind direction was significant (*p* < 0.001) in regression models of predictors of NO_x_, NO_2_, and NO.

### Health outcomes and their associations with residential proximity to traffic

Table 4 presents ORs for current asthma and bronchitis within the preceding 12 months with increasing residential traffic, within a 150-m radius, adjusted for important covariates. Overall, comparing the highest with the lowest quintiles, traffic density and maximum AADT were associated with increased ORs for current asthma. A test for trend with increasing quintiles of traffic was significant (*p* ≤0.05) for traffic density and current asthma. For bronchitis, we observed associations for the 90th percentile, with traffic density being statistically significant.

Using distance to major roads as an exposure metric, we found associations between current asthma (or bronchitis) and log distance to highways and for those within 75 m of highways (Table 4). Associations were elevated but not significant using distance to freeway/ highway on a linear scale. Those living downwind and within 300 m of a freeway/highway were at increased risk of both outcomes; however, results were not statistically significant, possibly due to small numbers in the higher exposure categories. We could not examine wind effects at fine cut-points because of limited sample size. To explore whether other major roads were associated with respiratory problems, we restricted our analysis to those participants who did not live within 150 m of a freeway/highway (*n* = 867). We found no clear associations between current asthma (or bronchitis) and living within 75 m of a principal artery among this subgroup (Table 4).

Our sensitivity analyses indicated that *a*) using a different measure of traffic density (distance-Gaussian-weighted traffic density) yielded results generally similar to those found using traffic density reported in Table 4 [see Supplemental Material, Table 3 (http://www.ehponline.org/members/2008/10735/suppl.pdf)]; *b*) associations using traffic metrics with buffer size of 300 m generated lower point estimates and wider CIs compared with the buffer sizes of 150 m; *c*) after restriction of the sample to those who lived at their current residence for at least 1 year, overall point estimates remained similar but with wider CIs because of smaller sample size; and *d*) we were unable to discern independent effects of school traffic exposure. When we added school-based concentration of BC or NO to multivariate models containing residential-based traffic, the effect estimate for residential traffic was mildly attenuated and no longer statistically significant. School-based NO and BC had borderline significance in the models (*p* < 0.12). Effect estimates for residential traffic were essentially unchanged with the addition of the school pollutant NO_2_, PM_10_, or PM_2.5_.

Our findings were robust to different questionnaire-based definitions of current asthma [see Supplemental Material, Table 4 (http://www.ehponline.org/members/2008/10735/suppl.pdf)]. In our stratified analysis, we found no clear difference in associations between current asthma or bronchitis and residential proximity to traffic when stratified by sex. When stratified by history of maternal asthma, we found that associations between traffic (log distance to freeway) and current asthma were higher among children without history of maternal asthma compared with those with a maternal history of asthma. Paternal history of asthma was not a risk factor or effect modifier for current asthma.

## Discussion

We demonstrated associations between residential proximity to traffic-related air pollution and current asthma using several indicators of nearby traffic. Additionally, an association was suggested between bronchitis symptoms in the preceding 12 months and traffic proximity at the highest levels of exposure. The traffic metrics we used in this study correlated with measured traffic pollutants, supporting their use. The traffic metric most weakly correlated with actual pollutant measurements (closest AADT) was not associated with respiratory symptoms.

This study adds to a growing body of evidence linking proximity to traffic and adverse respiratory effects. When we initiated this study, several studies, primarily in Europe, had identified associations between proximity to traffic and adverse respiratory outcomes [reviewed by Delfino (2002)]. However, extrapolations of the results of European studies to the United States is subject to a variety of sources of uncertainty, including differences in fleet composition (diesel vs. gasoline), emission controls, land use patterns, and population characteristics. Additionally, California has the most stringent emissions standards for motor vehicles in the United States. These differences could result in lower exposures to traffic pollutants among California residents relative to those in European cities.

Our study location and design allowed us to evaluate the effects of traffic pollution in a region of California with relatively low levels of regional air pollution. This restricted study area allowed us to focus on variations in air quality related to localized traffic-related air pollution. Our air monitoring pilot study confirmed that this small area variation in air quality was attributable to local impacts of traffic. Therefore, our study implicates local traffic as an important risk factor for respiratory disease in an urban area that meets federal air quality standards for ozone and annual average PM_2.5_ and has rare exceedances of the 24-hr PM_2.5_ standard. Other American studies of traffic and respiratory health involving populations from Southern California, the northeastern United States, and Anchorage, Alaska, had moderate to high regional levels of ozone and/or PM_2.5_ (English et al. 1999; Garshick et al. 2003; Gauderman et al. 2005; Lin et al. 2002; McConnell et al. 2006) or volatile organics from gasoline exhaust (Gordian et al. 2006). Thus, our study provides additional evidence that local traffic may have respiratory impacts even in an area with good regional air quality.

In the present study, we sought to reduce uncertainties related to exposure assessment in several ways. In our previous work, we reported modest effects using exposures assigned at a group level (based on neighborhood school measurements of traffic pollutants). In contrast, in this analysis, we found stronger associations using residential proximity to traffic, which may be attributable to less exposure misclassification. Also, we were able to evaluate and confirm the correlation between GIS-based indicators of traffic exposure and measured levels of traffic pollutants. As noted above, few epidemiologic studies relating respiratory health risks to traffic-related pollution have used actual pollution measurements or have validated their surrogate measures of traffic (Brauer et al. 2007; Gauderman et al. 2005; Hoek et al. 2002; Janssen et al. 2003; Nicolai et al. 2003). Finally, most traffic pollution models have not incorporated wind direction. Our study area has strong prevailing winds, and there was some suggestion that those living downwind of traffic might be at greater risk of respiratory symptoms, but results were not significant.

In addition to traffic metrics that used traffic counts within a given buffer, we also evaluated two simpler metrics based on distance and log distance of residences to busy roads (e.g., major artery or freeway). Our findings that children living within 75 m of a freeway/highway were at markedly increased risk of current asthma are consistent with studies in Massachusetts and Southern California that found elevated respiratory risks primarily among those living within the first 50–75 m of a busy road (Garshick et al. 2003; McConnell et al. 2006). In contrast, the same investigators in Southern California found, in a different cohort of children, that although the risk of asthma declined with increasing linear distance from a freeway, increased risks extending beyond several hundred meters (Gauderman et al. 2005). It is unclear whether the more linear decline in risks in the latter study were attributable only to direct impacts of freeway traffic emissions or whether other covariates (e.g., other major roads, area sources, and land use differences near freeways in urban areas) played an etiologic role. Additionally, McConnell et al. (2006) found an increased risk of asthma among those living near other major roads, whereas our results were less clear. The traffic volumes on some freeways and major roads in Southern California can be as much as double those experienced in the San Francisco Bay Area, which may explain the null findings in the current study.

Our study and several others have found that risks of either current or ever asthma are associated with proximity to traffic, and were elevated primarily among children with no reported family history of asthma (Gordian et al. 2006; McConnell et al. 2006) or maternal history of asthma (present study). Paternal history of asthma was not a risk factor or effect modifier for asthma in our study but may have been underreported by the parent respondent (6.6% reported a paternal history of asthma vs. 12.3% maternal history). The implication that children with no family history of asthma may be at increased risk for development or persistence of asthma from traffic-related pollutants deserves further investigation.

The cross-sectional nature of our study design is an important limitation of our study. Additional limitations include the relatively small sample size, the use of surrogates of exposure, and potential unmeasured confounders. In our previous study where we used school-based measurements, we found that modest effect estimates were slightly increased, and results became significant only after restricting analyses to those living at their current address as long-term residents, whereas in this analysis effect estimates remained similar. It is likely that the measurement error was greater when we used school-based measures, so restricting our analyses to long-term residents may have resulted in significant improvements in the estimates. In contrast, the residential metrics appear to have less measurement error, so less improvement in the estimates was gained by restricting analyses to long-term residents in this study.

Race/ethnicity and other socioeconomic status (SES) covariates were only weakly associated with current asthma in our study (crowding was a covariate in our final models), which may be attributable partly to our study design (i.e., we selected the schools to have relatively similar measures of SES profiles). Nonetheless, our results are consistent with several recent longitudinal studies in Europe and Southern California that have found associations between residential traffic and asthma incidence (Gauderman et al. 2005; McConnell et al. 2006).

Regarding exposure, we used measures of residential proximity to traffic as proxies for exposures to traffic-related pollution. Recent studies have found good correlations between personal exposures to traffic pollutants and residential proximity to traffic (Nethery et al. 2007; van Roosbroeck et al. 2006). In our study area, traffic pollution is likely to readily penetrate indoors, because this region experiences mild climate conditions, and the generally older housing stock tends not to have air conditioning or the degree of thermal insulation found in colder climates. However, the traffic metrics used in this study are surrogates for a complex mixture of traffic pollutants composed of reactive gases and PM, not just NO_x_. Many constituents of traffic exhaust may contribute to toxicity. For instance, human exposure studies have found that both PM_2.5_ in diesel exhaust and NO_2_ can enhance allergic responses (Barck et al. 2002; Riedl and Diaz-Sanchez 2005). Most epidemiologic investigations of traffic emissions, including ours, have not been designed to disentangle the relative contributions of diesel versus gasoline combustion. However, to the extent that our findings were strongly influenced by proximity to freeways, this suggests that something specific to freeway traffic (e.g., higher percentages of diesel trucks as well as high traffic volume) may be important.

It is interesting to note that in our study, NO_2_, a secondary product of traffic emissions, had stronger correlations with 300-m metrics than with 150-m metrics. However, traffic metrics at 300 m (traffic density within 300 m and maximum AADT within 300 m) had weaker associations with current asthma compared with the corresponding metric at 150 m. This may be purely a dose-related phenomenon, reflecting the exponential decay of pollutant concentrations with distance from freeways, or may suggest that “fresh” primary traffic emissions, such as ultrafine PM_0.1_, may be important determinants of the observed associations with current asthma. Although we did not design this study to look separately at the contribution of traffic at school versus home, nor was the sample size sufficient to do so, we saw some mild attenuation of residential traffic when we added study-averaged concentrations of BC or NO (but not NO_2_) to multivariate models, again suggesting that “fresh” primary emissions may be important constituents.

Our results contribute to a growing body of evidence linking residential proximity to traffic with the prevalence of respiratory symptoms and asthma in children. These findings are observed across diverse populations worldwide, despite differences in demographics, lifestyle, transportation patterns, and levels of regional air pollution. Although the identities of the constituents of traffic pollution most strongly linked with health impacts have yet to be elucidated, traffic emissions clearly have an adverse impact on both local and regional air quality and respiratory health. Reducing exposures to traffic pollution will provide a healthier environment for children where they live, play, and learn.

## Figures and Tables

**Table 1:** Traffic metrics used in exposure assessment.

Traffic metric^a^	Description	Reference
Maximum AADT within 150 m	Highest traffic count of any road within a 150-m radius	English et al. 1999
Closest AADT within 150 m	Traffic count of the closest nonlocal road within a 150-m radius	English et al. 1999
Traffic density	Vehicle miles traveled (VMT) within a 150-m radius of the residence: VMT = sum of [(bidirectional AADT) × (length of respective road segments)].	Gunier et al. 2003
Distance to major road	Different definitions of “major road” evaluated based on federal highway designations (e.g., interstates, highways, major arteries); we used natural logarithm of distance in some analyses	Gauderman et al. 2005

a We assigned local roads a value of zero. We also evaluated traffic metrics using a buffer radius of 300 m in the sensitivity analysis.

**Table 2:** Demographics, home characteristics, health status, and residential traffic exposures of study participants (*n* = 1,080).

Characteristic	Value
Sex (%)	
Percent female	52.3
Race/ethnicity (%)	
White	12.9
Black	11.0
Hispanic	43.3
Asian	13.7
Other/multiracial	19.2
Indicators of SES	
Household at/below federal poverty level (%)	31.4
Parent’s education, high school or less (%)	29.6
Crowding [no. people/bedroom (median)]	2
Family history (%)	
Mother with asthma	12.2
Maternal smoking during pregnancy	10.4
Home indoor environment (%)	
Smoker in the household, current	7.4
With furry pet in the house	37.2
With pests, preceding 12 months	63.1
With gas stove	63.2
With indicator of mold/mildew, preceding 12 months	44.8
Health characteristics (%)	
Ever asthma	19.7
Current asthma	11.5
Bronchitis in the preceding 12 months	12.4
Hay fever or allergic rhinitis	11.9
Chest illness before 2 years of age	23.5
Residential proximity to traffic [median (range)]	
Maximum AADT within 150 m^a^ (vehicles/day)	9,500 (0–245,000)
Closest AADT within 150 m^a^ (vehicles/day)	8,190 (0–245,000)
Traffic density within 150 m (vehicle-km traveled)	2,884 (0–74,042)
Distance to freeway/highway (m)	791 (22–3,671)
Distance to major road (principal artery, expressway, highway, or freeway) (m)	246 (7–996)
Percent living within 100 m of major road (principal artery or higher)	16.0
Percent living within 100 m of freeway/highway	5.0

a Local roads were assigned a value of zero.

**Table 3:** Spearman correlation (ρ) between GIS-based traffic metrics and traffic pollutants.

	NO_2_		NO_x_		NO	
Traffic metric	ρ	*p*-Value	ρ	*p*-Value	ρ	*p*-Value
Within 150 m						
Maximum AADT	0.14	0.325	0.37	0.006	0.43	0.001
Closest AADT	0.01	0.957	0.22	0.118	0.26	0.058
Traffic density	0.14	0.333	0.36	0.008	0.41	0.003
Distance to freeway/highway^a^	−0.30	0.028	−0.48	< 0.001	−0.69	< 0.001
Within 300 m						
Maximum AADT	0.38	0.006	0.56	< 0.001	0.60	< 0.001
Closest AADT	0.14	0.324	0.29	0.034	0.22	0.117
Traffic density	0.40	0.003	0.58	< 0.001	0.62	< 0.001

a Spearman correlations are same for natural-log distance to freeway.

**Table 4:** Associations between metrics of residential proximity to traffic and current asthma and bronchitis in the preceding 12 months.^a^

	OR (95% CI)	
Traffic metric	Current asthma (*n* = 88/724)	Bronchitis (*n* = 87/745)
Maximum AADT within 150 m (vehicles/day)		
1st quintile (local traffic only )	1.00	1.00
2nd quintile (up to 7,120)	1.50 (0.67–3.36)	0.93 (0.46–1.87)
3rd quintile (7,121–18,900)	2.33 (1.03–5.28)	1.02 (0.49–2.12)
4th quintile (18,901–28,657)	0.60 (0.21–1.69)	0.46 (0.19–1.12)
5th quintile ( 28,658–245,000)	2.50 (1.13–5.53)	1.42 (0.71–2.81)
≥90th percentile (67,000–245,000)	2.40 (1.13–5.07)	1.96 (0.97–3.95)
Closest AADT within 150 m (vehicles/day)		
1st quintile (local traffic only)	1.00	1.00
2nd quintile (up to 5,700)	1.39 (0.62–3.11)	0.77 (0.38–1.57)
3rd quintile (5,701–10,534)	2.83 (1.23–6.54)	1.40 (0.67–2.91)
4th quintile (10,535–23,800)	1.40 (0.60–3.29)	0.90 (0.43–1.86)
5th quintile (23,801–245,000)	1.58 (0.69–3.65)	0.90 (0.42–1.9)
≥90th percentile (35,100–245,000)	1.16 (0.53–2.54)	1.11 (0.52–2.33)
Traffic density within 150 m^b^		
1st quintile	1.00	1.00
2nd quintile	1.23 (0.53–2.83)	0.58 (0.27–1.25)
3rd quintile	1.96 (0.85–4.52)	1.47 (0.73–2.95)
4th quintile	1.40 (0.60–3.3)	0.78 (0.36–1.67)
5th quintile	2.37 (1.05–5.36)	1.16 (0.57–2.36)
≥90th percentile	2.14 (1.02–4.52)	2.12 (1.09–4.10)
Log distance to freeway/highway^c^	1.43 (1.04–1.54)	1.47 (1.11–1.96)
Distance to freeway/highway		
≤75 m	3.80 (1.20–11.71)	2.81 (0.94–8.39)
> 75 to ≤ 150 m	1.87 (0.71–4.90)	1.82 (0.75–4.40)
> 150 to ≤ 300 m	1.25 (0.50–3.11)	2.00 (0.93–4.29)
> 300 m	1.00	1.00
Distance to freeway/highway and wind orientation		
≤ 300 m, downwind	1.41 (0.81–2.46)	1.42 (0.87–2.33)
≤ 300 m, upwind	1.05 (0.58–1.91)	1.13 (0.66–1.95)
> 300 m	1.00	1.00
Distance to principal artery (excluding those near freeway/highway)^d^		
≤ 75 m	1.36 (0.51–3.62)	1.49 (0.61–3.67)
> 300 m	1.00	1.00

a ORs adjusted for crowding, pests, indicators of mold presence, and chest illness before 2 years of age. For asthma, we also adjusted models for maternal history of asthma.

b See Supplemental Material (http://www.ehponline.org/members/2008/10735/suppl.pdf) for quintile ranges.

c For distance to freeway (and log distance), ORs are for the interquartile ranges, that is, the difference between the 25th and 75th percentiles of residential distance from the freeway: 75th percentile (1,352 m) – 25th percentile (413 m).

d Includes only those participants living > 150 m of a freeway/highway (*n* = 980; median traffic counts on principal arteries were ~ 28,500 vehicles/day).
